# A Rare Presentation of Collision Diffuse Large B-Cell Lymphoma and Hodgkin Lymphoma Treated with R-CHOP and Adjuvant Radiation Therapy

**DOI:** 10.7759/cureus.52630

**Published:** 2024-01-20

**Authors:** Batoul Mazraani, Batoul Nasser, Bailey Loving, Zachary N Awad, Thomas J Quinn

**Affiliations:** 1 Radiation Oncology, Oakland University William Beaumont School of Medicine, Rochester Hills, USA; 2 Radiation Oncology, University of Michigan, Ann Arbor, USA; 3 Radiation Oncology, Beaumont Hospital, Royal Oak, USA

**Keywords:** adjuvant radiation therapy, r-chop, diffuse large b-cell lymphoma, non-hodgkin lymphoma, hodgkin lymphoma, malignant lymphoma, collision tumor

## Abstract

Collision tumors are rare neoplasms displaying two distinct cell populations developing in juxtaposition to one another without areas of intermingling. There are currently no guidelines for the recommended treatment for such rare collision cases. We herein report a unique case of a 45-year-old female who presented with a left-sided palpable inguinal lymph node. A subsequent excisional biopsy yielded a diagnosis of collision lymphoma (CL) of nodular sclerosing Hodgkin lymphoma (HL) and germinal center diffuse large B-cell lymphoma (DLBCL). This case report highlights the challenges in managing CL and the potential efficacy of cyclophosphamide, doxorubicin, vincristine, prednisone, and rituximab regimen (R-CHOP) and adjuvant radiation therapy (RT) in treating this rare condition. Our goal is to enrich the literature with our case on CL in an attempt to progress to a path of ultimately establishing a definitive treatment approach to CL of DLBCL and HL.

## Introduction

Malignant lymphomas have been divided into two distinct groups: Hodgkin lymphoma (HL) and non-Hodgkin lymphoma (NHL). NHL constitutes about 80% of all lymphomas, with the common ones being diffuse large B-cell lymphoma (DLBCL) and follicular lymphoma [1]. HL and NHL may occur sequentially or simultaneously in the same individual, and in rare cases, a clonal relationship between the lymphomas has been proven [2].

Upon clinical examination, patients with DLBCL and HL typically present with enlarging lymphadenopathy and constitutional symptoms such as fever, weight loss, and night sweats. Morphologically, DLBCL is characterized by a diffuse infiltration of medium-to-large cells with large nucleoli and abundant cytoplasm [3]. On the other hand, a Reed-Sternberg cell or lymphocyte-predominant cell needs to be identified within the biopsy specimen to establish a diagnosis of HL [4].

The chemotherapy regimen most commonly used for the treatment of DLBCL is rituximab, cyclophosphamide, doxorubicin, vincristine, and prednisone (R-CHOP) followed by adjuvant radiation therapy (RT), which leads to cure in approximately 50-60% of patients [3]. The standard chemotherapy regimen for HL is doxorubicin hydrochloride, bleomycin sulfate, vinblastine sulfate, and dacarbazine (ABVD) for most patients [4]. Positron emission tomography/computed tomography (PET/CT) is the standard test for the assessment of treatment response in lymphomas [4].

Collision lymphomas (CL) constitute the presence of two different types of NHL or a rare association of NHL and HL in a single organ or tissue. They are uncommon, with an incidence ranging from 1% to 4.7%, depending on the series [5]. Both composite and collision tumors involve two morphologically and immunohistochemically distinct neoplasms coexisting within a single organ. However, collision tumors lack the histological cellular intermingling seen in composite tumors. Composite tumors often arise from a common driver mutation that induces a divergent histology from a common neoplastic source while collision tumors may arise from coincidental neoplastic change [6]. One of the strongest predictors of long-term survival after DLBCL treatment is the maintenance of an event-free status until a minimum of 24 months after initial diagnosis similar to that of an age- and sex-matched general population [7]. This case illustrates a rare phenomenon of collision DLBCL and HL in a patient with no history of lymphoma that was treated with R-CHOP and adjuvant RT, with an expected excellent prognostic outcome.

## Case presentation

**Figure 1:**
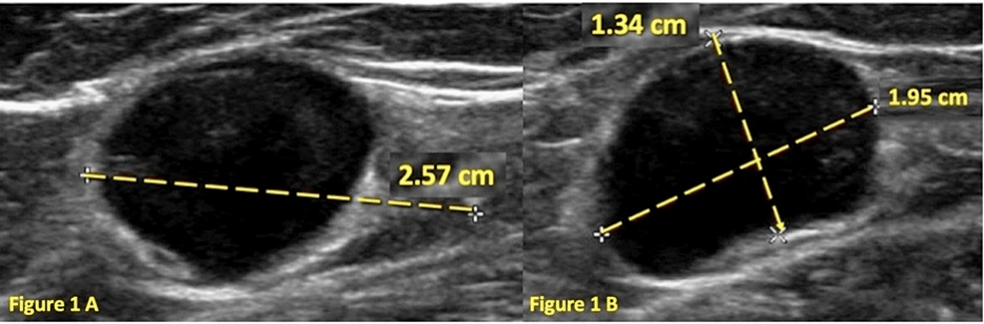
Short- and long-axis ultrasound of the left groin demonstrating an abnormally enlarged lymph node with abnormal morphology, measuring 2.57 cm x 1.95 cm x 1.34 cm with hypervascularity

A 45-year-old female with no significant past medical history presented with a palpable left inguinal lymph node. She reported associated fatigue over the past six months but otherwise denied weight loss, fever, chills, or night sweats. On physical examination, a non-tender, fairly immobile, 2 cm nodule was palpated in the left lower groin. An ultrasound of the left groin demonstrated an abnormally enlarged lymph node measuring 2.57 x 1.95 x 1.34 cm with hypervascularity (Figure 1). Excisional biopsy of the left groin lymph node yielded a diagnosis of both classical nodular sclerosing HL and germinal center DLBCL.

**Figure 2:**
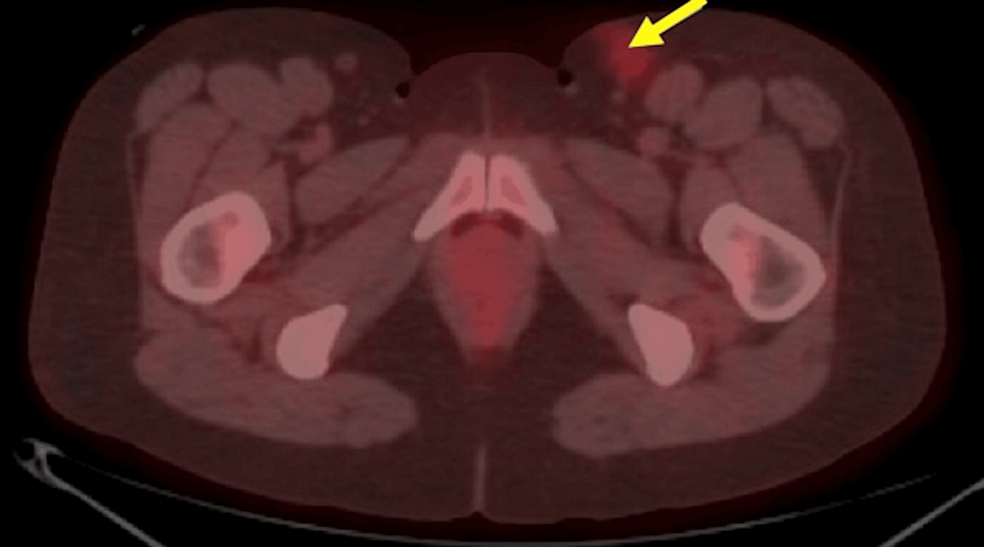
Axial PET/CT imaging demonstrating low-level tracer uptake at the presumed left inguinal excisional biopsy site (blue arrow) and no definite sites of hypermetabolic malignancy. PET/CT: positron emission tomography/computed tomography

Prior to treatment, immunohistochemical studies of the inguinal lymph node revealed Hodgkin/Reed-Sternberg (HRS) cells expressing antibodies to CD30, CD15, and variable CD20 and PAX-5. In contrast, the confluent areas of large cells show a different phenotype: uniform and strong CD20 expression along with PAX-5, CD10, BCL6, and BCL2. Of note, these areas were negative for CD30 and CD15, with an increased Ki-67 proliferation fraction of 50-60%. In addition, a bone marrow biopsy was obtained at the time and yielded negative results for malignancy. Initial PET/CT imaging demonstrated low-level tracer uptake at the site of inguinal excision with SUV max of 2.1, diffuse low-level marrow hypermetabolism, and no evidence of definite hypermetabolic malignancy (Figure 2). Mediastinal SUV maximum was 1.4 and hepatic parenchyma SUV maximum was 2.3. Positron emission tomography/computed tomography (PET/CT) performed after radiation treatment demonstrated minimal stranding of the subcutaneous fat within the left groin at the site of the previously visualized enlarged inguinal lymph node. At this time, the mediastinal SUV maximum was 1.6 and the hepatic parenchyma SUV maximum was 2.3.

Complete blood count (CBC), comprehensive metabolic panel (CMP), erythrocyte sedimentation rate (ESR), lactate dehydrogenase, haptoglobin, and serum beta-2 microglobulin levels were within normal limits. An acute hepatitis panel and antinuclear antibody screen yielded negative results. Immunoglobulin G kappa monoclonal protein and serum-free light chains were mildly elevated, with free Kappa at 2.21 mg/dL and a free Kappa/Lambda ratio of 2.10. These extensive investigations established a diagnosis of favorable stage IA CL of germinal center DLBCL and classical nodular sclerosing HL.

Given the scarcity of data and treatment guidelines on mixed neoplastic components, the decision was made to treat according to the DLBCL treatment paradigm. The patient completed three cycles of R-CHOP q21d. The rituximab was given concurrently with CHOP. The standard protocol for R-CHOP-21 is cyclophosphamide 750 mg/m^2^, doxorubicin 50 mg/m^2^, vincristine 1.4 mg/m^2^ (maximum dose 2 mg), and rituximab 375 mg/m^2^. The patient dosage was calculated with adjustments for the patient’s BSA value of 1.6 m^2^. Following the calculations, the dosage given to the patient was 600 mg of rituximab, 1260 mg of cyclophosphamide, 80 mg of doxorubicin, 2 mg of vincristine, and 100 mg of prednisone. The patient developed headaches, nausea, and fatigue, which were most prominent approximately four to eight days after her infusions and resolved after the completion of her chemotherapy regimen.

**Figure 3:**
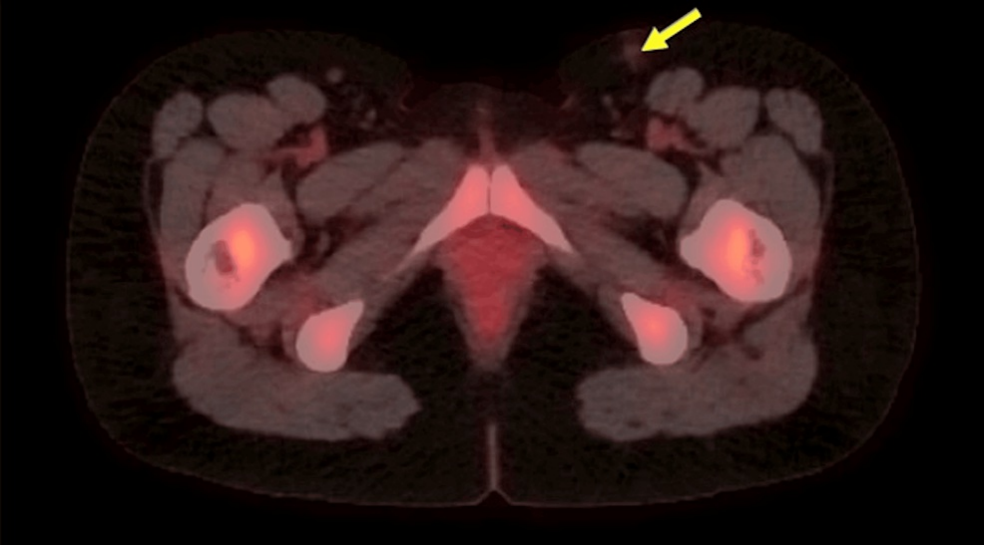
Axial PET/CT imaging demonstrated no evidence of new, persistent, or recurrent malignant disease. A previously FDG-avid lymph node in the left inguinal region has resolved nearly to the background (blue arrow) PET/CT: positron emission tomography/computed tomography; FDG: fluorodeoxyglucose

PET/CT imaging was performed after treatment and demonstrated no evidence of persistent or recurrent malignant disease. There was almost complete resolution of the fluorodeoxyglucose (FDG) active lymph node seen in the left inguinal region on the last PET/CT (Figure 3). These results demonstrated a complete response to systemic therapy. The patient then proceeded to receive 14 Gray (Gy) in 7 fractions of three-dimensional-conformal radiation therapy (3D-CRT) and 16 Gy in 8 fractions of volumetric modulated arc therapy (VMAT) for a total of 30 Gy in 15 fractions over an 18-day span. The patient tolerated the symptoms well. There were two different types of treatment due to insurance initially denying VMAT. The first seven fractions were delivered with 3D-CRT to ensure no delay in care. The last eight fractions were given with VMAT once insurance finally approved the modality.

On one-month follow-up post-RT, the patient had returned to normal activity and felt well overall, with no symptoms noted. The patient will be reassessed with repeat PET/CT imaging every few months, with an expected excellent prognostic outcome.

## Discussion

The presence of a CL of HL and DLBCL, which are two morphologically and clinically distinct neoplasms, is rare. Due to the diversity and complexity of lymphomas and the varied causes of different lymphoma types, no mechanisms have been available to explain the pathogenesis of different types of CL. Little literature exists discussing a similar occurrence of HL and NHL in a single node or multiple nodes [8].

In a literature review by Trecourt et al., patients with a composite lymphoma of classic Hodgkin’s lymphoma and DLBCL are classically old or middle-aged, without a history of lymphoma at presentation [9]. Similarly, our patient had no past medical history of lymphoma. Collision tumors currently have no guidelines for management and treatment focuses primarily on the predominant tumor. In the literature review by Wang et al, the most commonly utilized treatment modalities consisted of either CHOP or R-CHOP [10].

There has been a case previously reported by Rosenquist et al. in 2004, which demonstrated the presence of a CL with HL and DLBCL in an inguinal lymph node. In that case, the presence of a CL was demonstrated through histological examination. Following surgical excision of the lymph node, the patient was treated with six cycles of R-CHOP. The patient responded well with subtotal regression of the lymphoid masses and peripheral blood count that remained within normal limits. At follow-up 12 months after diagnosis, the patient had excellent clinical outcomes with no evidence of disease recurrence or progression [11]. Our patient is one of the few reported cases of collision DLBCL and HL coexisting in an inguinal lymph node.

Researchers are swinging the pendulum toward the idea that HL and B-cell NHL coexisting in the same tissue might originate from a common precursor [12]. Prior observations have demonstrated that the nodular lymphocyte-predominant HL subtype most commonly transforms into more aggressive lymphomas, namely, DLBCL [13,14]. The hypothesis of the transformation of HL to DLBCL is a consideration; however, the patient's overall histopathological features do not entirely support large B-cell transformation given the patient’s nodular sclerosing variant of HL. In addition, the two distinct types of lymphomas occurred simultaneously in the inguinal node at the time of diagnosis as opposed to a more indolent lymphoma preceding the aggressive form.

In regards to treatment, our approach focused on addressing the more aggressive component, DLBCL, of the CL while simultaneously achieving remission in the more indolent component. R-CHOP has become the standard of care for patients with newly diagnosed DLBCL [15]. Following three cycles of R-CHOP, the patient’s PET/CT imaging demonstrated no evidence of persistent or recurrent disease. A retrospective study by McLaughlin et al. demonstrated a survival benefit with the use of consolidation RT for early-stage DLBCL, which prompted adjuvant RT following the utilization of R-CHOP [16].

The fact that ABVD, not R-CHOP, is the standard therapeutic regimen used for patients with HL raises the question of whether or not R-CHOP is enough to control the progression of HL. Although standardized approaches to the treatment of DLBCL and nodular sclerosing HL as separate entities exist, the optimal therapeutic approach for the CL presented here is unknown. In up to 20-30% of patients with cHL, Reed-Sternberg (RS) cells express CD20 [17]. Anti-CD20 monoclonal antibody rituximab may be an effective therapeutic strategy in cHL, both by direct killing of RS cells and by targeting the surrounding microenvironment [18,19]. Thus, we anticipate that the administration of R-CHOP may confer some protection against disease progression of HL.

Mediastinal gray zone lymphoma (MGZL) is related to the phenomenon of collision tumors [20]. MGZL presents with primary mediastinal large B-cell lymphoma, a subtype of DLBCL, along with overlapping features of the nodular sclerosing subtype of classical HL [20]. The optimal therapy for MGZL is controversial and unknown; however, it has been proposed that a regimen of dose-adjusted etoposide, prednisone, vincristine, cyclophosphamide, doxorubicin, and rituximab (DA-EPOCH-R) may be a reasonable approach [20]. Future studies should investigate the efficacy of DA-EPOCH-R in cases such as ours.

## Conclusions

In conclusion, we were not able to find a clinical case in the literature identical in all aspects to our CL case. This is a rare case of collision lymphoma of the germinal center subtype of diffuse large B-cell lymphoma (DLBCL) and classical nodular sclerosing Hodgkin lymphoma (HL) presenting in a single lymph node. DLBCL and HL can originate from a common precursor cell through somatic mutations, but this is commonly seen in the nodular lymphocyte-predominant HL subtype. Understanding the histological background of CL could be the first step in identifying the pathophysiology of the disease. Our goal is to enrich the literature with our case on CL in an attempt to progress to a path of ultimately establishing a definitive treatment approach to CL of DLBCL and HL.
